# Revisiting sea-level budget by considering all potential impact factors for global mean sea-level change estimation

**DOI:** 10.1038/s41598-022-14173-2

**Published:** 2022-06-17

**Authors:** Fengwei Wang, Yunzhong Shen, Qiujie Chen, Jianhua Geng

**Affiliations:** 1 State Key Laboratory of Marine Geology, Tongji University, Shanghai, People’s Republic of China; 2 College of Surveying and Geo-Informatics, Tongji University, Shanghai, People’s Republic of China; 3 School of Ocean and Earth Science, Tongji University, Shanghai, People’s Republic of China

**Keywords:** Physical oceanography, Climate change, Physical oceanography

## Abstract

Accurate estimates of global sea-level change from the observations of Altimetry, Argo and Gravity Recovery and Climate Experiment (GRACE) and GRACE Follow-on (GRACE-FO) are of great value for investigating the global sea-level budget. In this study, we analyzed the global sea-level change over the period from January 2005 to December 2019 by considering all potential impact factors, i.e. three factors for Altimetry observations (two Altimetry products, ocean bottom deformation (OBD) and glacial isostatic adjustment (GIA)), three factors for Argo observations (four Argo products, salinity product error and deep-ocean steric sea-level change), and seven factors for GRACE/GRACE-FO observations including three official RL06 solutions, five spatial filtering methods, three GIA models, two C_20_ (degree 2 order 0) products, Geocenter motion, GAD field and global mass conservation. The seven impact factors of GRACE/GRACE-FO observations lead to ninety combinations for the post-procession of global mean barystatic sea-level change estimation, whose rates range from 2.00 to 2.45 mm/year. The total uncertainty of global barystatic sea-level change rate is ± 0.27 mm/year at the 95% confidence level, estimated as the standard deviation of the differences between the different datasets constituting the ensembles. The statistical results show that the preferred GIA model developed by Caron et al. in 2018 can improve the closure of the global sea-level budget by 0.20–0.30 mm/year, which is comparable with that of neglecting the halosteric component. About 30.8% of total combinations (GRACE/GRACE-FO plus Argo) can close the global sea-level budget within 1-sigma (0.23 mm/year) of Altimetry observations, 88.9% within 2-sigma. Once the adopted factors including GRACE/GRACE-FO solutions from Center for Space Research (CSR), Caron18 GIA model, SWENSON filtering and Argo product from China Second Institute of Oceanography, the linear trend of global sterodynamic sea-level change derived from GRACE/GRACE-FO plus Argo observations is 3.85 ± 0.14 mm/year, nearly closed to 3.90 ± 0.23 mm/year of Altimetry observations.

## Introduction

Since 1993, the global mean sea level (GMSL) has risen more than 8 cm with a rate of 3.30 mm/year estimated from satellite Altimetry observations with inverse barometer corrections, called sterodynamic sea-level change^1^, which is dominated by two components: the ocean mass redistribution and steric changes^2,3^. About one-third of the global sea-level rise can be attributed to ocean thermal expansion and salinity changes (steric component) that can be measured down to 2000 m depth by the Argo project since 2005, the rest (barystatic component) is attributed to the ocean mass contribution related to ice sheets, glaciers and land water storage^2,4^, which can be measured by Gravity Recovery and Climate Experiment (GRACE) and GRACE Follow-on (GRACE-FO) missions launched in 2002 and 2018, respectively. Therefore the global sea-level budget can be expressed as follows^2,5^,

1 $$GMSL_{{^{sterodynamic} }} = GMSL_{steric} + GMSL_{{^{barystatic} }} ,$$

where, $GMSL_{{^{sterodynamic} }}$ is the global mean sterodynamic sea-level change, $GMSL_{{^{steric} }}$ is the steric component including thermosteric and halosteric contributions and $GMSL_{{^{barystatic} }}$ is the barystatic component. Considering that the global averaged halosteric contribution should be essentially zero due to the salinity conservation^1,6,7^, many studies only consider the thermosteric sea-level change to investigate the global sea-level budget. Besides, since the ocean thermal expansion changes are only measured for the upper 2000 m of the ocean by Argo floats and the ocean mass change will cause Ocean Bottom Deformation (OBD), the OBD and deep-ocean thermosteric sea-level change (> 2000 m) should be considered when studying the global sea-level budget^8,9^. Then the updated global sea-level budget equation is expressed as follows,

2 $$GMSL_{{^{sterodynamic} }} - GMSL_{OBD} = GMSL_{thermosteric} + GMSL_{thermosteric \, (deep)} + GMSL_{{^{barystatic} }} ,$$

where $GMSL_{OBD}$ denotes the effect of OBD and $GMSL_{{thermosteric{ (}deep{)}}}$ is the deep-ocean thermosteric sea-level change.

Many previous publications have tried to close the global sea-level budget using different datasets and post-processed strategies over different periods^10–15^, however, the misclosure is quite different (Table 1). From Table 1, we can clearly find that adopting different Altimetry products will lead to certain differences in estimated global mean sterodynamic sea-level change rates, for example, 3.11 ± 0.24 mm/year over 2005–2015^9^, 3.50 ± 0.20 mm/year over 2005–2015^2^ and 3.79 ± 0.18 mm/year over 2005–2015^16^, similarly for both RL06 solutions of GRACE/GRACE-FO and Argo observations. Recently published studies have shown that the global sea-level budget can be closed within the respective uncertainties during the GRACE era, however, appears no longer closed after 2016^15^, even though only thermosteric sea-level change is used to prevail the problem of fast salinity drift that some Argo floats are experiencing since 2016^17^.

**Table 1:** Published estimates of global mean sea-level change rates from Altimetry, Argo, GRACE and GRACE-FO solutions [mm/year].

GMSL rates	Time-span	Sterodynamic (Altimetry)	Barystatic (GRACE GRACE-FO)	Steric (Argo)	Note
Chambers et al.^11^	2005.1–2014.12	3.17 ± 0.67	2.11 ± 0.36	0.97 ± 0.15	Steric
Dieng et al.^10^	2004.1–2015.12	3.49 ± 0.14	2.24 ± 0.10	1.14 ± 0.09	Steric
WCRP Global Sea Level Budget Group.^2^	2005.1–2015.12	3.50 ± 0.20	2.30 ± 0.19	1.30 ± 0.40	Thermosteric
Chen et al. ^16^	2005.1–2015.12	3.79 ± 0.18	2.61 ± 0.14	1.11 ± 0.10	Steric
Vishwakarma et al.^9^	2005.1–2015.12	3.11 ± 0.24	1.63 ± 0.20	1.22 ± 0.12	Steric
Chen et al.^14^	2005.1–2016.12	3.87 ± 0.16	2.39 ± 0.16	1.12 ± 0.08	Steric
Wang et al.^18^	2005.1–2016.12	3.76 ± 0.12	2.43 ± 0.14	1.16 ± 0.08	Steric
Chen et al.^15^	2005.1–2020.4	3.92 ± 0.30	2.22 ± 0.10	1.00 ± 0.22	Steric
Barnoud et al.^17^	2005.1–2019.12	3.96 ± 0.23	2.14 ± 0.02	1.31 ± 0.05	Thermosteric

All the statistical results are part results of these above references. Steric includes thermosteric and halosteric contributions and Thermosteric includes only thermosteric contribution.

**Figure 1:**
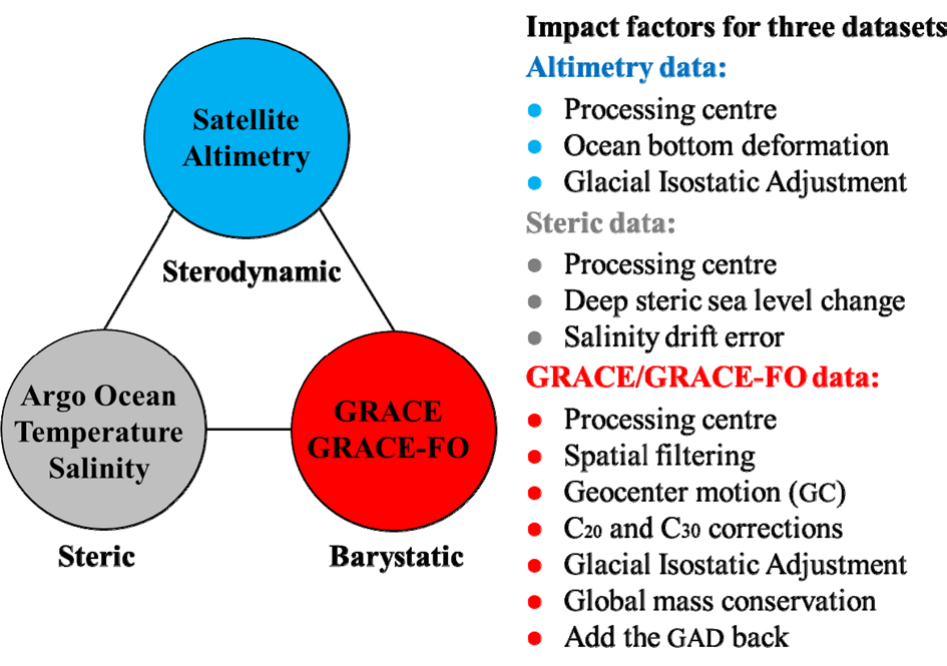
The impact factors of global sea-level change estimated from Altimetry, Argo and RL06 solutions of GRACE/GRACE-FO observations.

Compared to Altimetry and Argo observations, accurate quantification of global mean barystatic sea-level changes from RL06 solutions of GRACE/GRACE-FO observations has been more challenging because more factors affect the quantification^6,14,15,18–20^, for example, spatial filtering for reducing strong noise, replacing the C_20_ (degree 2 order 0) coefficients of RL06 solutions of GRACE/GRACE-FO observations with that from Satellite Laser Ranging (SLR)^21^, Geocenter motion correction (GC, degree-1 SH coefficients) and Glacial Isostatic Adjustment (GIA) correction^22,23^. Chen et al.^14^ investigated the four impact factors on global mean barystatic sea-level change estimates and found that there exists a linear trend misclosure from 0.36 to 0.58 mm/year relative to Altimetry minus Argo estimates over the period from January 2005 to December 2016, and a systematic annual phase lag between GRACE/GRACE-FO and Altimetry minus Argo estimates due to the enforced mass conservation in RL06 solutions of GRACE/GRACE-FO observations^16^. However, to correctly implement global mass conservation, the negative of the GAC C_00_ coefficients (representing the total mass of the atmosphere) should be added to RL06 solutions of GRACE/GRACE-FO observations^16^. Note that GAC is the GRACE supplementary data product of gravity change caused by global non-tidal atmospheric and high-frequency oceanic mass changes. Besides, for ocean applications, the GAD (i.e., GAD-GRACE supplementary data product of gravity change over the ocean caused by non-tidal atmospheric surface pressure and high-frequency oceanic mass changes) should be added back after its monthly ocean average is removed^7,24^, which are normally neglected due to the slight linear trend difference of global mean barystatic sea-level change. In summary, all potential impact factors of global sea-level change estimates are listed in Fig. 1.

Though some impact factors have been investigated for studying the global sea-level budget in previous publications^7,14,17,19,20^, not all factors among Altimetry, Argo and GRACE/GRACE-FO observations are analyzed simultaneously. In this contribution, we will take all potential impact factors into account to estimate the global mean sterodynamic, thermosteric and barystatic sea-level changes more accurately and rigorously, and further investigate the closure of the global sea-level budget. The rest of this paper is organized as follows: “Data and processing strategies” section briefly introduces the Data and Processing methods of this study. “Impact factors for global sea-level change estimation” section analyzes the impact factors for estimating global sea-level change using RL06 solutions of GRACE/GRACE-FO, Altimetry and Argo observations and finally, the concluding remarks and discussions are presented in “Conclusions and discussions” section.

## Data and processing strategies

### GRACE and GRACE-FO data

We adopt the RL06 solutions of GRACE/GRACE-FO observations provided by the Center for Space Research (CSR), German Geoforschungszentrum (GFZ) and Jet Propulsion Laboratory (JPL) covering the period from January 2005 to December 2019 with 30 months missing data to estimate global mean barystatic sea-level changes. The spherical harmonics (SH) coefficients of the RL06 solutions are truncated to the degree and order 60 and centered with the mean-field of the study period. Two SLR C_20_ coefficients (CSR SLR and GSFC SLR Technical Note 14 (TN-14)) are used to replace the C_20_ coefficients of RL06 solutions of GRACE and GRACE-FO observations^21,25^. Since the RL06 solutions do not contain degree-1 (C_11_, S_11_, C_10_) coefficients and the longest SLR degree-1 coefficients are just until 2017, only independent estimates of CSR GRACE Technical Note 13 (TN-13)^26^ are used for geocenter motion correction. Three GIA models are used to evaluate the impacts on global mean barystatic sea-level change estimates, mainly including ICE6G-D^27^, Caron18^28^ and A13^29^. A 300-km Gaussian smoothing and five different decorrelation filter methods are used to filter the strong noise, specifically including SWENSON^30^, P4M6^31^, P4M15^32^, DUAN^33^ and DDK1^34^. To reduce the land signal leakage^35^, we compute the global mean barystatic sea-level change by averaging global ocean grid points farther than 300 km from the coast with the latitudes from 64.5° S to 64.5° N (to be consistent with Altimetry and Argo latitude sampling discussed later), with the area weighting which can be approximately replaced by cosine latitude weighting. The time series of GRACE/GRACE-FO, Altimetry and Argo observations are all fitted with least-squares fitting by introducing the linear trend, annual and semi-annual terms. Note that the periods of 161 days and 3.73 years are removed for correcting the S2 and K2 ocean tide aliasing^18^.

### Altimetry and Argo data

Two different estimates of the Altimetry-based global gridded sea-level anomaly products are used in this study: (a) the 0.25° × 0.25° daily Altimetry Sea Surface Height (SSH) data, provided by the Archiving, Validation, and Interpretation of Satellite Oceanographic (AVISO), are averaged into monthly intervals to compute the global sterodynamic sea-level change; (b) the 0.25° × 0.25° daily gridded SSH data named SEALEVEL_GLO_PHY_CLIMATE_L4_MY_008_057, which are provided by the (CMEMS) Copernicus Marine Environment Monitoring Service. The global mean sterodynamic sea-level change is computed over global oceans farther than 300 km from the coast between the latitudes of 64.5° S and 64.5° N. Normally the GIA impacts for Altimetry observations are corrected by adding a constant value of − 0.30 mm/year^36^. Considering the study area in this study, the recomputed mean GIA impact is − 0.28 mm/year by using the ICE5G-VM2 model that is downloaded from https://www.atmosp.physics.utoronto.ca/~peltier/data.php.

The shallow steric sea-level change (< 2000 m) is determined from four gridded subsurface Argo ${1}^\circ \times 1^\circ$ products (including temperature, salinity and pressure datasets), which are provided by the International Pacific Research Center (IPRC), the Scripps Institute of Oceanography (SIO), Japan Agency for Marin-Earth Science and Technology (JAMSTEC) and China Second Institute of Oceanography (CSIO) respectively. Steric sea-level change is computed by using the Argo products as^37^,

3 $$SL_{{{\text{steric}}}} = - \frac{1}{{\rho_{0} }} \cdot \int_{ - h}^{0} {\Delta \rho \cdot dz} ,$$

where, $\rho_{0}$ is the mean density of seawater (1027 kg/m^3^), and $\Delta \rho$ is the density change as a function of temperature, salinity and pressure, which can be computed using the United Nations Educational, Scientific and Cultural Organization (UNESCO) standard equations^38^. Since the mean salinity is used in Eq. (3), any salinity effect on steric sea-level change is not considered here and we only focus on the thermosteric contribution. Then the global mean thermosteric sea-level change is computed with four Argo products over global oceans farther than 300 km from the coast between the latitudes from 64.5° S to 64.5° N. For the deep-ocean thermosteric component to the global mean separately, normally taking an estimate of 0.10 mm/year^2,9^, here in this study we adopt 0.12 ± 0.03 mm/year according to the estimated result of Chang et al.^39^.

## Impact factors for global sea-level change estimation

### Global sterodynamic sea-level change from Altimetry observations

**Figure 2:**
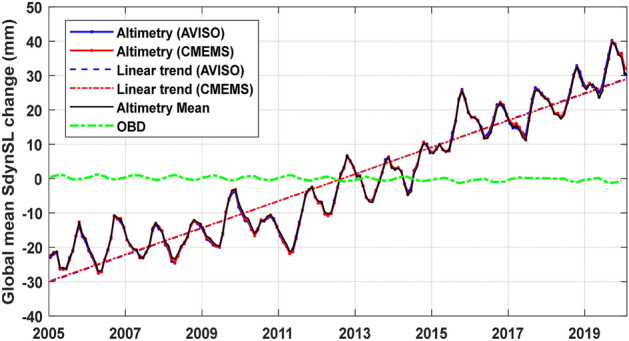
The global mean sterodynamic sea-level change series over January 2005 to December 2019 with two Altimetry products.

The sterodynamic sea-level change that equates to a sum of barystatic and thermosteric sea-level change^40^ can be directly estimated by satellite Altimetry since 1993^5^. The global mean sterodynamic sea-level changes estimated from two satellite Altimetry products over the period from January 2005 to December 2019 are presented in Fig. 2, which agree well with each other, with an average trend of 3.87 ± 0.23 mm/year after correcting the GIA effect by adding a constant of 0.28 mm/year. The same uncertainty (0.23 mm/year) as Barnoud et al.^17^ was estimated by taking into account all sources of errors affecting the Altimetry-based global mean sterodynamic sea-level change estimation^41^ over the same study period. Considering there exist 30 missing months in GRACE/GRACE-FO RL06 solutions over 2005–2019, after the same missing months are deleted from Altimetry observations, the re-estimated global mean sterodynamic sea-level change rate is 3.82 ± 0.23 mm/year, with a slight difference of 0.05 mm/year, the corresponding statistical results are presented in Table 2. Besides, OBD should be corrected due to the changes in ocean mass load^8^, which are computed using the RL06 solutions following the method of Vishwakarma et al.^9^. The linear trend of the global mean OBD series is − 0.08 ± 0.01 mm/year over the period from January 2005 to December 2019.

**Table 2:** Amplitudes of annual and semiannual components and linear trends of global mean sterodynamic sea-level change from Altimetry observations for the period January 2005 to December 2019.

Index	Annual amplitude [mm] phase [deg]	Semi-annual amplitude [mm] phase [deg]	Linear trend [mm/year]
Altimetry (AVISO)	[5.16 ± 0.48] [297.4 ± 5.3]	[1.14 ± 0.48] [162.8 ± 23.9]	3.89 ± 0.23
Altimetry (CMEMS)	[5.30 ± 0.47] [300.7 ± 5.1]	[1.26 ± 0.47] [162.3 ± 21.4]	3.85 ± 0.23
Altimetry mean	[5.24 ± 0.47] [301.1 ± 5.1]	[1.36 ± 0.47] [163.4 ± 19.7]	3.87 ± 0.23
Altimetry mean*	[5.12 ± 0.53] [300.9 ± 5.9]	[1.17 ± 0.52] [145.4 ± 25.3]	3.82 ± 0.23
OBD	[0.63 ± 0.02] [97.3 ± 1.7]	[0.09 ± 0.02] [256.3 ± 12.6]	− 0.08 ± 0.01
Altimetry mean*-OBD	[5.70 ± 0.54] [298.4 ± 5.3]	[1.21 ± 0.53] [141.6 ± 23.7]	3.90 ± 0.23

The uncertainty represents the least-squares fitting error (1 sigma for amplitudes, phases and trends) except the linear trend (± 0.23 mm/year) estimated as Barnoud et al.^17^. Same missing months as GRACE/GRACE-FO data are deleted from Altimetry data, the corresponding re-estimated results are given as (*).

### Global thermosteric sea-level change from Argo observations

**Figure 3:**
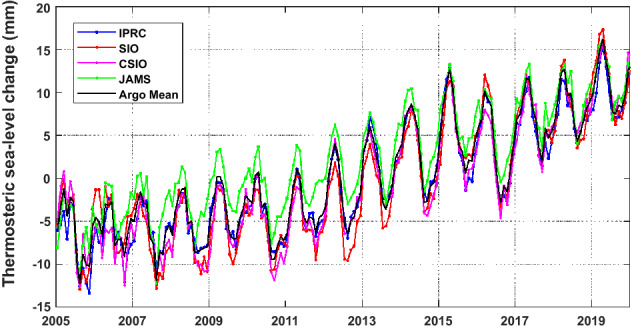
The global mean thermosteric sea-level change derived from four Argo products. The ensemble mean of four components is displayed as a black line.

Four Argo products (IPRC, SIO, CSIO and JAMSTEC) are used to compute the global mean thermosteric sea-level change. Due to the salinity conservation over global oceans, we neglect the halosteric component so as to avoid underestimating the linear trend because of the fast salinity drift error after 2016^17^. Figure 3 shows the global mean thermosteric sea-level changes derived from four Argo products, we can find that Argo-based estimates of IPRC, SIO and CSIO agree well with each other, with slight difference relative to that from JAMSTEC. After deleting 30 missing months same as GRACE/GRACE-FO solutions, the re-estimated global mean thermosteric sea-level change rate is 1.28 ± 0.04 mm/year nearly equal to 1.27 ± 0.04 mm/year as that before deleting missing months, but annual and semi-annual amplitudes are with slight differences (Table 3). Besides, the deep-ocean thermosteric sea-level change (> 2000 m) should be added for Argo observations^9^, whose trend contribution is 0.12 ± 0.03 mm/year^39^, the updated global mean thermosteric sea-level change rate is 1.40 ± 0.05 mm/year over the period from January 2005 and December 2019.

**Table 3:** Amplitudes of annual and semiannual components and linear trends of global mean thermosteric sea-level change from four Argo observations for the period January 2005 to December 2019.

Index	Annual amplitude [mm] phase [deg]	Semi-annual amplitude [mm] phase [deg]	Linear trend [mm/year]
Argo (SIO)	[4.71 ± 0.30] [84.1 ± 3.6]	[0.91 ± 0.30] [236.4 ± 19.0]	1.31 ± 0.05
Argo (IPRC)	[4.14 ± 0.22] [91.3 ± 3.1]	[0.74 ± 0.22] [225.4 ± 32.6]	1.26 ± 0.04
Argo (CSIO)	[4.26 ± 0.29] [88.9 ± 3.9]	[0.74 ± 0.29] [224.2 ± 6.7]	1.31 ± 0.05
Argo (JAMSTEC)	[3.80 ± 0.19] [78.5 ± 2.8]	[1.32 ± 0.19] [247.2 ± 8.2]	1.20 ± 0.03
Argo mean	[4.21 ± 0.22] [85.8 ± 3.0]	[0.91 ± 0.22] [235.6 ± 14.2]	1.27 ± 0.04
Argo mean*	[4.18 ± 0.25] [84.5 ± 3.4]	[0.96 ± 0.25] [239.9 ± 14.9]	1.28 ± 0.04
Deep thermosteric	–	–	0.12 ± 0.03
Argo mean* + deep thermosteric	[4.18 ± 0.25] [84.5 ± 3.4]	[0.96 ± 0.25] [239.9 ± 14.9]	1.40 ± 0.05

The uncertainty represents the least-squares fitting error (1 sigma for amplitudes, phases and trends). The same missing months as GRACE/GRACE-FO data are deleted from Argo observations, the corresponding re-estimated results are given as (*).

### Global barystatic sea-level change from GRACE/GRACE-FO gravity field solutions

From “Global sterodynamic sea-level change from Altimetry observations” and “Global thermosteric sea-level change from Argo observations” sections, the global mean sterodynamic and thermosteric change series are derived from Altimetry and Argo observations after corresponding impact factors (GIA, OBD, Deep-ocean thermosteric sea-level change) being corrected over the period from January 2005 to December 2019. In this subsection, we will estimate the global mean barystatic sea-level changes using the RL06 solutions of GRACE/GRACE-FO observations. As mentioned in “Introduction” section, the barystatic sea-level change estimates from GRACE/GRACE-FO RL06 solutions are easily affected by the adopted different post-processing strategies, mainly including three official processing centers (CSR, GFZ, JPL), Geocenter motion (GRACE TN-13), two SLR C_20_ coefficients (CSR SLR and GSFC SLR TN-14), SLR C_30_ (degree 3 order 0) coefficients (GSFC SLR TN-14), five decorrelation filter methods (P4M6, P4M15, SWENSON (P3M6), DUAN and DDK1), Gaussian smoothing (300 km), signal leakage correction (300 km buffer zone), three GIA models (A13, ICE6G-D and Caron18). These post-processing strategies will lead to ninety combined solutions of global mean barystatic sea-level change estimates, with which we can investigate the effect of these factors on estimating global mean barystatic sea-level changes and closing the global sea-level budget.

#### RL06 solutions from three official processing centers

**Figure 4:**
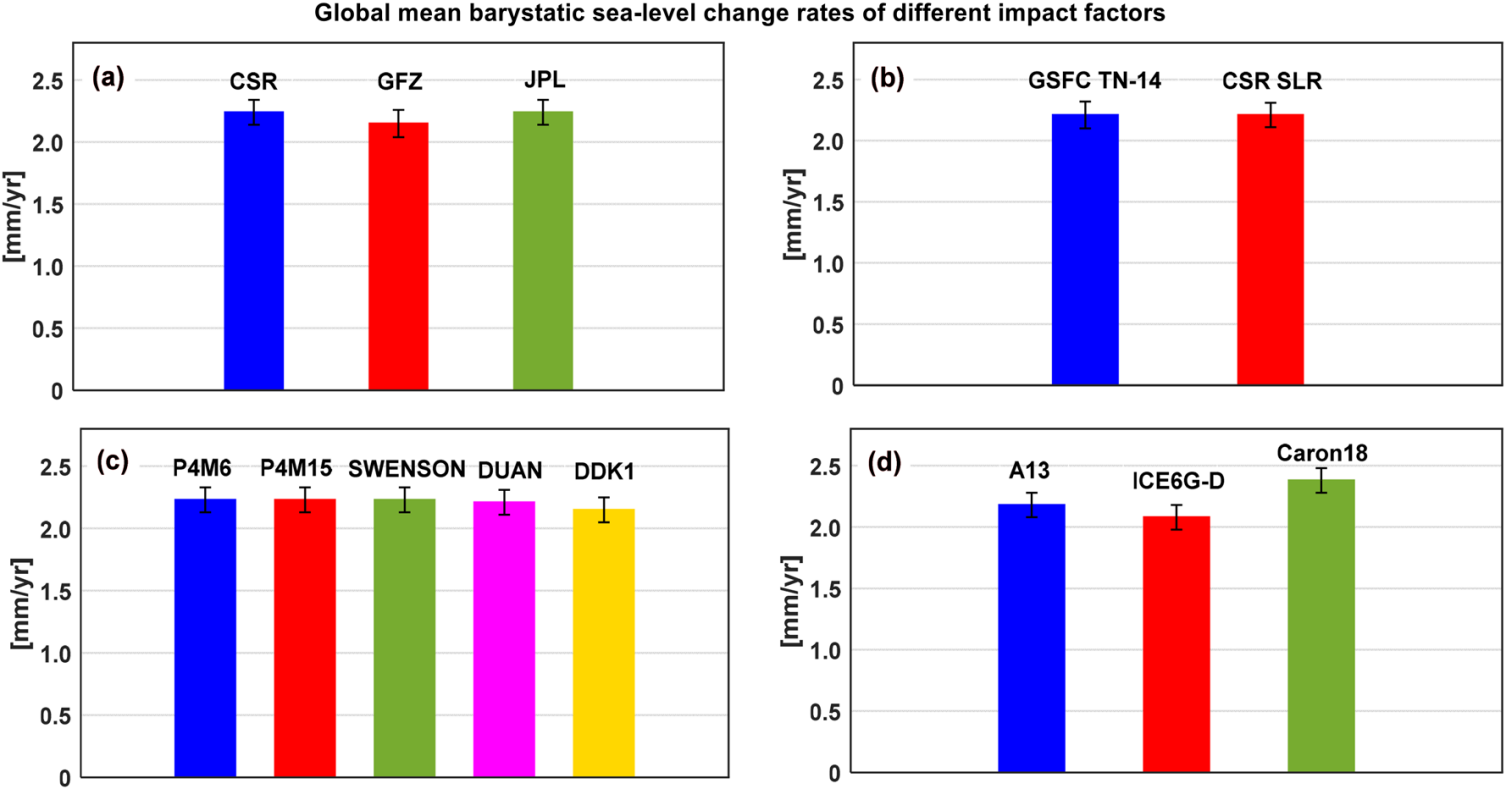
The linear trends of four potential impact factors on global mean barystatic sea-level change estimations over the study period. (**a**) Processing center, (**b**) C_20_ correction, (**c**) Filtering method and (**d**) GIA correction.

Different processing strategies of GRACE/GRACE-FO solutions will bring certain differences in estimating global mean barystatic sea-level change. Three RL06 solutions from different official processing centers are used to estimate the global mean barystatic sea-level change rate over the period from January 2005 to December 2019 in the open ocean mass farther than 300 km from the coast between the latitudes from 64.5° S to 64.5° N. Figure 4a shows the global mean barystatic sea-level change series and linear trends of three official RL06 solutions computed with the global mean barystatic sea-level changes of ninety combined solutions, in which the largest linear trend differences reach 0.09 mm/year (Table 4).

**Table 4:** The sub-ensemble mean amplitudes of annual and semiannual components and linear trends of global mean barystatic sea-level changes from GRACE/GRACE-FO solutions over January 2005 to December 2019.

Impact factors		Annual amplitude [mm] phase [deg]	Semi-annual amplitude [mm] phase [deg]	Linear trend [mm/year]
Processing Center	CSR (30)	[9.51 ± 0.32] [280.6 ± 1.9]	[0.79 ± 0.31] [66.4 ± 23.6]	2.24 ± 0.05
	GFZ (30)	[9.65 ± 0.33] [280.2 ± 1.9]	[0.90 ± 0.33] [36.8 ± 20.3]	2.15 ± 0.06
	JPL (30)	[9.54 ± 0.31] [280.4 ± 1.8]	[0.72 ± 0.31] [58.2 ± 20.1]	2.24 ± 0.05
C20 correction	TN14 (45)	[9.68 ± 0.33] [276.7 ± 1.9]	[0.72 ± 0.33] [65.2 ± 25.4]	2.21 ± 0.06
	CSR SLR (45)	[9.45 ± 0.31] [284.1 ± 1.8]	[0.88 ± 0.30] [42.4 ± 17.3]	2.21 ± 0.05
Filtering method	P4M6 (18)	[9.55 ± 0.32] [280.2 ± 1.9]	[0.80 ± 0.32] [52.8 ± 19.3]	2.23 ± 0.05
	P5M12 (18)	[9.67 ± 0.32] [279.9 ± 1.9]	[0.80 ± 0.32] [51.5 ± 20.2]	2.23 ± 0.05
	Swenson (18)	[9.23 ± 0.31] [281.0 ± 1.9]	[0.82 ± 0.31] [60.1 ± 22.9]	2.23 ± 0.05
	DUAN (18)	[9.77 ± 0.32] [280.2 ± 1.9]	[0.82 ± 0.32] [53.6 ± 22.3]	2.21 ± 0.05
	DDK1 (18)	[9.62 ± 0.31] [280.6 ± 1.8]	[0.78 ± 0.31] [50.9 ± 22.1]	2.15 ± 0.05
GIA correction	A13 (30)	[9.57 ± 0.32] [280.4 ± 1.9]	[0.80 ± 0.32] [53.8 ± 21.4]	2.18 ± 0.05
	ICE6G-D (30)	[9.57 ± 0.32] [280.4 ± 1.9]	[0.80 ± 0.32] [53.8 ± 21.4]	2.08 ± 0.05
	Caron18 (30)	[9.57 ± 0.32] [280.4 ± 1.9]	[0.80 ± 0.32] [53.8 ± 21.4]	2.38 ± 0.05

The (30), (45) and (18) represent the corresponding ensemble solutions. The uncertainty of each single impact factor ensemble (1 sigma for amplitudes, phases and trends).

#### C_20_ coefficients correction

Due to the limited ability of GRACE/GRACE-FO for estimating C_20_ coefficients, two different SLR C_20_ products (CSR SLR vs GSFC SLR TN14) are adopted to replace the GRACE/GRACE-FO C_20_ coefficients over the period from January 2005 to December 2019, the results indicating that two SLR C_20_ products have the same effects on global mean barystatic sea-level change rate and slight differences in the amplitudes and phases of annual and semi-annual components, the statistical results are presented in Fig. 4b and Table 4.

#### Spatial filtering methods

The applied different decorrelation filtering method has a small effect on the estimated global mean barystatic sea-level changes at both seasonal and long-term time scales. The averaged global mean barystatic sea-level change rates related to different filtering methods with all possible combined solutions range from 2.15 ± 0.05 to 2.23 ± 0.05 mm/year (Fig. 4c). However, except for the filtering method of DDK1 (2.15 ± 0.05 mm/year), the differences in the global mean barystatic sea-level change rates are less than 0.02 mm/year among the other four filtering methods. When no filtering is applied, the global mean barystatic sea-level change rate is 2.32 ± 0.05 mm/year, slightly larger than those of all filtering methods (Table 4).

#### GIA correction

The GIA signal induces significant trends in GRACE solutions that must be removed, which is a crucial factor for estimating barystatic sea-level change from GRACE/GRACE-FO observations. The only way to correct this signal is to use GIA models. However, different groups have independently developed GIA model solutions based on the Toronto ice history reconstruction, by using different implementations of GIA codes and somehow different Earth models, GIA models significantly differ^2^. We corrected the gravity field effect of GIA-related mass redistributions by using three different GIA modelling results: the model namely A13 by A et al.^29^ based on ICE-5Gv2 glaciation history from Peltier^42^; the model ICE-6G_D (VM5A) by Peltier et al.^27,43^; and the mean solution Caron18 by Caron et al.^28^, with the linear trends of − 1.10 mm/year, − 1.00 mm/year and − 1.30 mm/year respectively, which accounts for about 50% of the linear trend of total global mean barystatic sea-level change. It is obvious to find that when the GIA model of Caron et al.^28^ is used, the global mean barystatic sea-level change rate is higher (2.38 ± 0.05 mm/year) than the A13 and ICE6G-D models (2.18 ± 0.05 and 2.08 ± 0.05 mm/year), the corresponding results are presented in Fig. 4d and Table 4. Our preferred GIA model is the Caron18, which is based on the ICE-6G deglaciation history^43^, while the model by A et al.^29^ is based on its predecessor model, ICE-5G. Besides, the A13 and ICE6G-D models are single GIA models, the Caron18 model arises as a weighted mean from a large ensemble of models, where the glaciation history and the solid-Earth rheology have been varied and validated against independent geodetic data^44^.

#### Other impact factors

The atmospheric and oceanic masses (i.e., the so-called GAD product) are further needed to be added back but with the GAD mean over the ocean removed following the method of Uebbing et al.^7^. For full global barystatic sea-level change, it leads to very similar estimates with a slight difference (0.03 mm/year) for global mean barystatic sea-level change rates from January 2005 to December 2019 regardless of whether adding the GAD field back or not, however, has a certain effect on the annual and semi-annual amplitudes. There is an important issue that should be addressed is that since the CSR SLR and GSFC TN-14 C_20_ and C_30_ coefficients are estimated by restoring the GAD back, obviously, the GAD C_20_ and C_30_ coefficients should be ignored to avoid the problem of “double counting” even though the impact is relatively small. Besides, the global mass conservation correction is also corrected following the method of Chen et al.^16^.

#### Total uncertainty

In previous subsections of “Global barystatic sea-level change from GRACE/GRACE-FO gravity field solutions” section, we mainly analyze the impact factors (including processing center, C_20_, the filtering method, and the GIA correction) for estimating global mean barystatic sea-level change to determine to which extent the factors can bias GRACE/GRACE-FO estimates and compute an ensemble of ninety combinations for GRACE/GRACE-FO post-processing. The total uncertainties are estimated as the standard deviation of the differences between the different datasets constituting the ensembles^20^. This approach is likely to underestimate the uncertainty as it only considers the variability of a limited number of datasets^17^. Table 4 summarizes the sources of uncertainty in the GRACE/GRACE-FO estimate of the global barystatic sea-level change associated with each impact factor. In contrast to the global barystatic sea-level change rates, values vary greatly and depend significantly on the chosen factors. Based on our ensemble, the total uncertainty of the global mean barystatic sea-level change rate is ± 0.27 mm/year and comparable to that of Blazquez et al.^19^ from 2005 to 2019 at the 95% confidence level when assuming a Gaussian distribution, with the mean linear trend of 2.21 ± 0.14 mm/year. Though the spread of our ensemble does certainly not represent the true uncertainty in GRACE/GRACE-FO, it can be used to evaluate the total uncertainty of all impact factors for global mean barystatic sea-level change estimation to some extent.

### Global sea-level budget from Altimetry, Argo and GRACE/GRACE-FO observations

After estimating the global mean sterodynamic, thermosteric and barystatic sea-level changes from Altimetry, Argo and RL06 solutions of GRACE/GRACE-FO observations, the global sea-level budget can be investigated based on the sea-level budget Eq. (2). From “Global sterodynamic sea-level change from Altimetry observations”, “Global thermosteric sea-level change from Argo observations” and “Global barystatic sea-level change from GRACE/GRACE-FO gravity field solutions” sections, the averaged linear trends of global mean sterodynamic and thermosteric sea-level changes are 3.90 ± 0.23 mm/year and 1.40 ± 0.05 mm/year after correcting the corresponding impact factors over the study period. When the global barystatic sea-level changes from GRACE/GRACE-FO observations plus the Argo-based thermosteric sea-level change, it gives an alternative estimate of the global sterodynamic sea-level change with an uncertainty of ± 0.28 mm/year over the period from 2005 to 2019 derived from an ensemble of the total 360 combinations (at the 95 percent confidence level assuming that the uncertainty from GRACE/GRACE-FO and Argo are independent).

**Figure 5:**
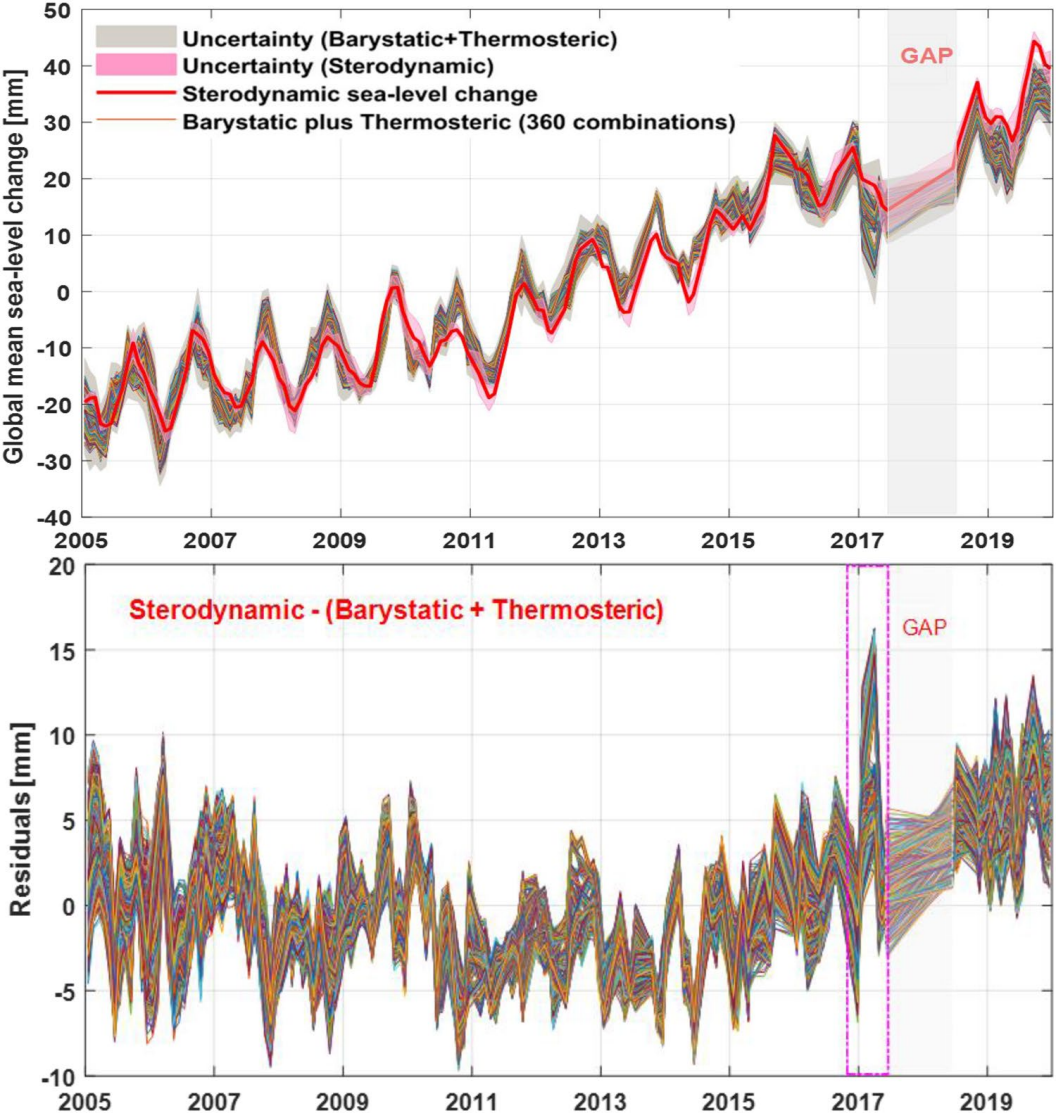
The global mean sterodynamic and sea-level change estimation from Altimetry and Argo plus GRACE and GRACE-FO observations and corresponding differences from 2005 to 2019.

Figure 5 shows the global mean sterodynamic sea-level change estimation from Altimetry and GRACE/GRACE-FO plus Argo observations over the period January 2005 to December 2019. The average linear trends are 3.61 ± 0.14 mm/year (Barystatic plus Thermosteric) and 3.90 ± 0.23 mm/year (Altimetry), respectively. From Fig. 5, it is obvious to find that the GRACE data differ around 2017 (shown in pink box of bottom subfigure) and there exist some systematic differences since 2016, mainly due to the problems in the accelerometer instrument leading to the increased errors in GRACE gravity solutions^44^.

**Figure 6:**
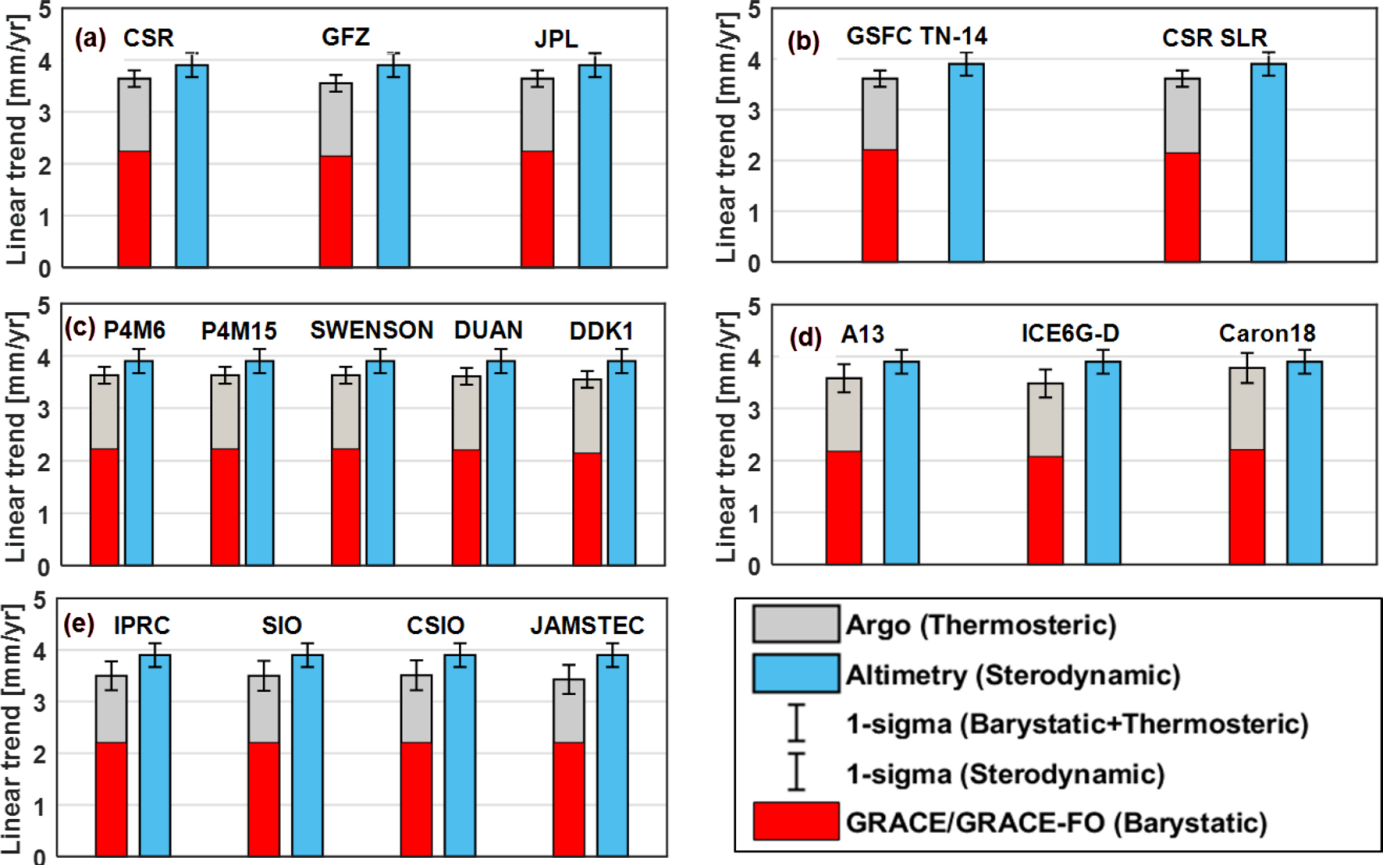
Linear trends of the global sea-level budget over January 2005 to December 2019 for Altimetry, Argo and GRACE/GRACE-FO observations over the sub-ensembles according to (**a**) processing center, (**b**) C_20_ correction, (**c**) filtering method, (**d**) GIA correction and (**e**) Argo product.

Besides, we further present the linear trends of the global sea-level budget from January 2005 to December 2019 for Altimetry, Argo and GRACE/GRACE-FO observations over the sub-ensembles with five main factors (processing center, filtering method, GIA model, C_20_ correction and Argo product) in Fig. 6. For the linear trend, about 30.8% of 360 combinations (barystatic plus thermosteric) can close the global sea-level budget within 1-sigma of Altimetry observations if the adopted factors include the Caron18 GIA model and 88.9% within 2-sigma. When the impact factors are CSR (processing center), CSR SLR C_20_, SWENSON (filtering method), Caron18 GIA model and CSIO (Argo), respectively, the largest global mean sterodynamic (barystatic plus thermosteric) sea-level change rate among 360 combinations is 3.85 ± 0.14 mm/year, nearly closed to the 3.90 ± 0.23 of Altimetry observations, which are consistent with the results of Tables 3 and 4.

## Conclusions and discussions

There exist many impact factors for investigating the global sea-level budget using the GRACE/GRACE-FO RL06 solutions, Altimetry and Argo observations. It is normally recognized that the sea-level budget can be closed within the uncertainty at a global scale, however, failed to be closed since 2016, seems have a systematic difference between the global mean sterodynamic sea-level change obtained by the Altimetry and GRACE/GRACE-FO plus Argo data^17,45,46^. Through the comparison of comprehensive experiments, we take all potential impact factors (shown in Fig. 1) into account to evaluate their effect on the closure of the global sea-level budget from 2005 to 2019. The new results from Altimetry show substantially larger global mean sterodynamic sea-level change rates than those of GRACE/GRACE-FO plus Argo observations, which are comparable to similar estimates from previous studies (as summarized in Table 1). For global barystatic sea-level change estimation, GIA correction has a more remarkable effect than other impact factors, whereas the preferred Caron18 GIA model can contribute more to improve the closure of the global sea-level budget than the A13 and ICE6G-D models. The ensemble-mean global barystatic sea-level change rate is 2.21 mm/year with a total uncertainty of ± 0.27 mm/year at a 95% confidence level estimated with the different datasets constituting the ensemble of ninety post-processed combined solutions.

Due to the fast salinity drift error of some Argo data after 2016, which introduced a negative trend leading to the underestimation of steric sea-level change. The re-assessment global sea-level budget just with the thermosteric sea-level change component can decrease the misclosure ~ 0.30 mm/year. We use four Argo products (IPRC, SIO, CSIO and JAMSTEC) to estimate global thermosteric sea-level change, with a mean linear trend of 1.40 ± 0.05 mm/year after adding the deep-ocean thermosteric contribution of 0.12 ± 0.03 mm/year estimated by Chang et al.^39^. After considering all potential impact factors, the updated results show that the linear trends of 360 combinations of global sterodynamic sea-level change derived from GRACE/GRACE-FO plus Argo range from 3.40 ± 0.28 to 3.85 ± 0.28 mm/year, consistent with 3.90 ± 0.46 mm/year of Altimetry observations at a 95 percent confidence level. Considering that the GRACE-FO mission has been observed about four years, further accumulation of observation data will be better to investigate the remaining misclosure of global sea-level budget among three independent observation systems.

## Data Availability

The merged Mean Sea Level Anomalies (MSLA) from TOPEX/Poseidon, Jason-1/2, ERS-1/2, and Envisat observations are provided by the Archiving, Validation, and Interpretation of Satellite Oceanographic (AVISO) data (http://www.aviso.oceanobs.com/). The 0.25° × 0.25° daily Altimetry SSH data are downloaded from the website of CMEMS https://resources.marine.copernicus.eu/. The RL06 GRACE/GRACE-FO gravity field solutions are downloaded from the International Centre for Global Earth Models website (http://icgem.gfz-potsdam.de/series/01_GRACE/CSR). Two Geocenter motion series of CSR GRACE Technical Note 13 geocenter motion series is downloaded from https://podaac-tools.jpl.nasa.gov/. The CSR SLR C_20_ spherical harmonics coefficients (http://download.csr.utexas.edu/pub/slr/degree_2/) and GSFC SLR Technical Note 14 C_20_ (https://podaac-tools.jpl.nasa.gov/) are used to replace GRACE/GRACE-FO C_20_ coefficients. Besides, GRACE Technical Note 14 C_30_ coefficients are also used to replace the GRACE-FO C_30_ coefficients, which are available at the website of https://podaac-tools.jpl.nasa.gov/. Three GIA models are adopted to evaluate their impacts on global mean barystatic sea-level change, including ICE6G-D model from http://www.atmosp.physics.utoronto.ca/∼peltier/data.php; Caron18 model (https://vesl.jpl.nasa.gov/solid-earth/gia/), A13 ftp://podaac-ftp.jpl.nasa.gov/allData/tellus/L3/pgr/.
